# A Comprehensive Survey of Genomic Mutations in Breast Cancer Reveals Recurrent Neoantigens as Potential Therapeutic Targets

**DOI:** 10.3389/fonc.2022.786438

**Published:** 2022-03-21

**Authors:** Si Zhou, Songming Liu, Lijian Zhao, Hai-Xi Sun

**Affiliations:** ^1^ College of Life Sciences, University of Chinese Academy of Sciences, Beijing, China; ^2^ College of Medical Technology, Hebei Medical University, Shijiazhuang, China

**Keywords:** breast cancer, neoantigens, immunotherapy, *PIK3CA*, SNVs

## Abstract

Neoantigens are mutated antigens specifically generated by cancer cells but absent in normal cells. With high specificity and immunogenicity, neoantigens are considered as an ideal target for immunotherapy. This study was aimed to investigate the signature of neoantigens in breast cancer. Somatic mutations, including SNVs and indels, were obtained from cBioPortal of 5991 breast cancer patients. 738 non-silent somatic variants present in at least 3 patients for neoantigen prediction were selected. *PIK3CA* (38%), the highly mutated gene in breast cancer, could produce the highest number of neoantigens per gene. Some pan-cancer hotspot mutations, such as *PIK3CA* E545K (6.93%), could be recognized by at least one HLA molecule. Since there are more SNVs than indels in breast cancer, SNVs are the major source of neoantigens. Patients with hormone receptor-positive or HER2 negative are more competent to produce neoantigens. Age, but not the clinical stage, is a significant contributory factor of neoantigen production. We believe a detailed description of breast cancer neoantigen signatures could contribute to neoantigen-based immunotherapy development.

## Introduction

Breast cancer is the most commonly diagnosed cancer in women worldwide (1). More than two million new breast cancer cases in 2018 contributed to one-fourth of women cancers (2). Breast cancer is a highly heterogeneous tumor that is currently classified by three molecular markers, including estrogen receptor (ER), progesterone receptor (PR) and HER2 (also called *ERBB2*). Treatment methods and prognosis of different breast cancer subtypes vary considerably (3, 4).

In recent years, cancer immunotherapy played an important role in a variety of solid tumors (5–7). The most representative immunotherapy approach is immune checkpoint blockade (ICB), but ICB therapy is only about 30 percent effective (8). Neoantigens exist specifically in tumor cells with better specificity and safety (9), and require major histocompatibility complexes (MHCs) to be recognized by immune cells to activate anti-tumor immune responses. Neoantigen-based immunotherapy can present a wide range of potential targets *via* MHC molecules presenting neoantigens (10), which is complementary to ICB therapy, such as neoantigen-based tumor-infiltrating lymphocytes (TILs) therapy in metastatic breast cancer (11). However, Tumors have a variety of immune escape mechanisms and high heterogeneity, with differences in tumor variation between different subtypes and even between individual patients. The limitation of neoantigen-based immunotherapy is that there are fewer neoantigens shared among different patients, and neoantigen-based therapeutics may be affected by immune checkpoints. Combining neoantigen and immune checkpoint inhibition therapy or chemoradiotherapy may achieve better therapeutic effects (12). T-cell immunotherapy based on *KRAS* K12D mutation has been reported in colorectal cancer (13), but similar therapies have not been reported in breast cancer.

HLA (Human Leukocyte Antigen) is a 3.6Mb segment of the human genome at 6p21.3 (14). There are two classical types of HLA: HLA-I and HLA-II. HLA-I molecules are responsible for antigen recognition and presentation, making them vital in neoantigen-based immunotherapy. HLA-II molecules, which present extracellular antigens, are also crucial to the human immune system.

With the development of sequencing technology, more and more studies on the mutation characteristics of breast cancer based on second-generation sequencing technology have been published (15–18). Here we focus on common neoantigens derived from high frequency mutations to benefit as many patients as possible. By integrating clinical information and mutation data of the 8 previous breast cancer research cohorts, we obtained the mutational landscape of 5991 breast cancer patients (4, 15–19). Finally, combining the high-frequency HLA information and mutation data, we got the most common shared neoantigens in breast cancer patients, which provides a new road for neoantigen-based immunotherapy.

## Results

### The Mutation Landscape of Breast Cancer Patients

**Figure 1:**
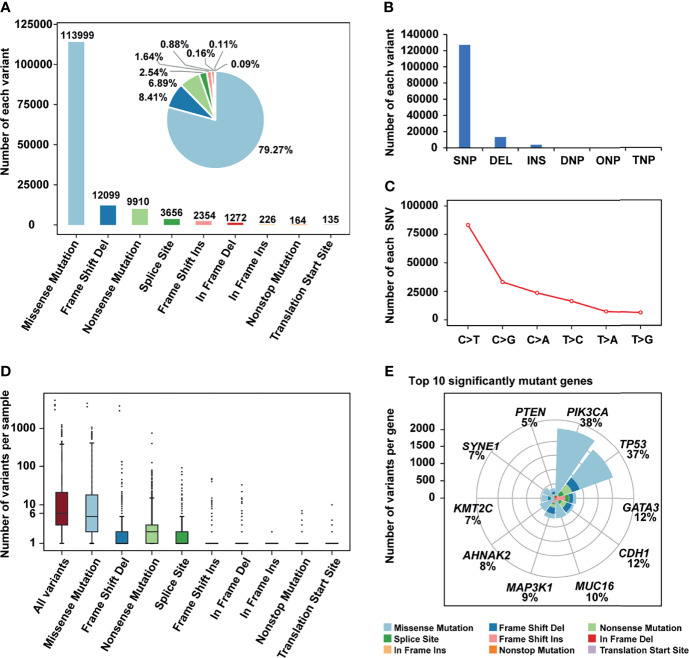
The mutation landscape of breast cancer cohort. **(A)** Bar plot and pie plot showing the number of each variant; **(B)** Bar plot and pie plot showing the number of each variant type; **(C)** Line graph showing the number of each SNV class; **(D)** Boxplot showing the number of variants per sample, the median of mutations per patient is 6; **(E)** Top 10 significantly mutant genes and the composition of variants.

The mutation status of all breast cancer samples was shown in **Figure 1** and **Supplementary Figure 1**. Missense mutation was the main variant classification. At base substitution level, C>T transition was the most common mutation event (**Supplementary Figures 2A–C**). Besides, the mutation load of each sample was relatively low, with only 25.3 mutations per patient on average and 6 mutations for the median. *PIK3CA* (38%) and *TP53* (37%) were two significantly mutated genes, with frequencies higher than others, such as *GATA3* (12%) and *CDH1* (12%) (**Figure 1F**). Many cancer-causing genes are co-occurring or show strong exclusiveness. This kind of interaction was also observed in our breast cancer cohort (**Supplementary Figure 2D**). For instance, *PIK3CA* and *TP53* were mutually exclusive while *PIK3CA* and *CDH1* were co-occurrent.

Although *TP53* and *PIK3CA* mutations frequently occurred regardless of the HER2 status, the mutated rates differed. In HER2^+^ patients, *TP53* (66%) mutated more frequently than *PIK3CA* (32%), while the mutated rates of *TP53* and *PIK3CA* were 33% and 40% in HER2^-^ patients (**Supplementary Figure 3**). In further investigation, we identified 22 differentially mutated genes between these two subgroups (Fisher’s exact test, *P* < 0.01, **Supplementary Figure 4**). The same analysis was also carried out in breast cancer patients with different HR (Hormone Receptor) statuses (**Supplementary Figure 5**).

Specially, we described the mutation status of triple-negative breast cancer (TNBC) patients. In our cohort, 70% of HR^-^ (ER^-^/HR^-^) patients were triple-negative breast cancer, leading to a high consistency of their mutation landscape (**Supplementary Figures 6A-F**). *TP53* mutations, which differentially happened between TNBC and non-TNBC patients, were observed in 79% of triple-negative breast cancer patients in our cohort (**Supplementary Figure 6G**).

### Results of Neoantigen Prediction

Due to the difference in the frequency of HLA in different populations, high-frequency (> 5%) HLA genotypes were selected from Han Chinese (20) and Americans (21) to predict “public” neoantigens (**Supplementary Table 1**).

After filtering, there were 617 eligible SNVs and 121 eligible indels, producing 356 and 86 derived peptides respectively (**Supplementary Tables 2**, **3**). In terms of SNVs, mutations of *PIK3CA*, *AKT1*, *SF3B1*, and *ESR1* produced the top 10 neoantigens with the highest frequency (**Table 1**), especially for *PIK3CA*, occupying 6 of 10. As for indels (**Table 2**), although the mutation frequency was lower, the number of neoantigens per mutation was higher, 2.69 for each indel but only 1.34 for each SNV on average.

**Table 1:** Top 10 SNVs and corresponding neoantigens.

Chr	Location	Gene	AA change	Peptide	Frequency	HLA types
chr3	178952085	PIK3CA	H1047R	ARHGGWTTK	839	HLA-B27:05
chr3	178936091	PIK3CA	E545K	ITKQEKDFLW	415	HLA-B57:01
chr3	178936091	PIK3CA	E545K	STRDPLSEITK	415	HLA-A03:01; HLA-A11:01
chr14	105246551	AKT1	E17K	RGKYIKTWR	196	HLA-A31:01
chr3	178921553	PIK3CA	N345K	ATYVKVNIR	132	HLA-A31:01
chr2	198266834	SF3B1	K700E	QEVRTISAL	83	HLA-B40:01
chr2	198266834	SF3B1	K700E	GLVDEQQEV	83	HLA-A02:01
chr3	178938934	PIK3CA	E726K	KTQKVQMKF	64	HLA-A32:01; HLA-B57:01
chr6	152419926	ESR1	D538G	LYGLLLEML	59	HLA-A24:02
chr3	178927980	PIK3CA	C420R	KEEHRPLAW	48	HLA-B44:03

**Table 2:** Top 10 indels and corresponding neoantigens.

Chr	Location	Gene	AA change	Peptide	Frequency	HLA types
chr3	178916938	PIK3CA	E110del	KVIEPVGNREK	11	HLA-A03:01; HLA-A11:01
chr5	56177011	MAP3K1	R763Cfs*35	LMFHKLSL	8	HLA-B08:01
chr5	56177011	MAP3K1	R763Cfs*35	FLLNFILIL	8	HLA-A02:01; HLA-A02:07
chr5	56177011	MAP3K1	R763Cfs*35	LILSVLMFH	8	HLA-A03:01
chr5	56177011	MAP3K1	R763Cfs*35	NFLLNFILI	8	HLA-A24:02
chr5	56155721	MAP3K1	R273Sfs*27	KSFPSAFSEW	7	HLA-B57:01
chr5	56155721	MAP3K1	R273Sfs*27	TTPKSPFTR	7	HLA-A11:01; HLA-A68:01
chr5	67591104	PIK3R1	K567_L570del	KRMNSIIQLR	7	HLA-B27:05
chr5	56155721	MAP3K1	R273Sfs*27	SPFTRWLL	7	HLA-B08:01
chr5	67591104	PIK3R1	K567_L570del	RMNSIIQLR	7	HLA-A31:01

### Comparison of Neoantigens in Different Subgroups

**Figure 2:**
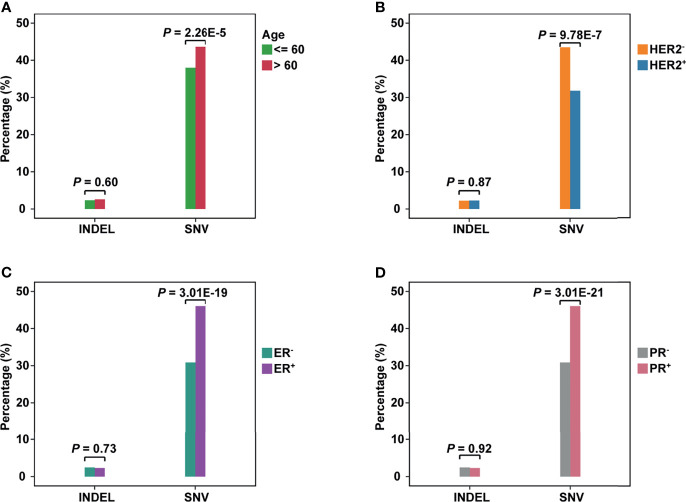
The comparison of neoantigens between different subgroups of breast cancer. **(A–D)** The horizontal axis represents the neoantigens source, including INDELs and SNVs; the vertical axis represents the percentage of neoantigen-carrying patients in the corresponding subgroup. **(A)**: Group Age: <=60 *vs >*60; **(B)** Group HER2 status: HER2^+^ *vs* HER2^-^; **(C)** Group ER status: ER^+^ *vs* ER^-^; **(D)** Group PR status: PR^+^ *vs* PR^-^.

Patients were divided into different subgroups by several clinical characteristics to investigate the relations between clinical information and neoantigens. By comparing the fraction of neoantigen-carrying patients in the corresponding subgroup, we found a higher fraction of the elderly population carrying SNV-derived neoantigens than younger ones (Fisher’s exact test, *P* = 2.26e-5, **Figure 2A**). As for the results of ER or PR status subgroups, the proportion of patients carrying SNV-derived neoantigens was higher in positive patients (**Figures 2C, D**). On the contrary, neoantigens of SNVs were more likely to be produced by HER2- patients (**Figure 2B**). No significant difference was observed in indel-derived neoantigens.

To evaluate the influence of SNV background within each subgroup, we compared the number of non-synonym SNVs in patients (**Supplementary Figure 7A**). For the age subgroup, the elderly population carried more SNVs (Wilcoxon test, *P* = 0.021). This may be why the elderly population is easier to produce neoantigens. As for ER or PR status, negative patients held a higher background. However, negative patients showed a lower non-synonym SNV load in the HER2 subgroup.

The clinical-stage was unlikely to be a critical factor in neoantigen production. Although we have observed the difference between patients in Stage I and Stage III (Fisher’s exact test, *P* = 0.005, **Supplementary Figure 7B**), the difference in other stages was not statistically significant. Compared to indels, SNV-derived neoantigens could cover more patients no matter in which subgroup (Wilcoxon test, *P =* 1.6e-5, **Supplementary Figure 7C**).

### Hotspot Mutations Derived Neoantigens May Serve as Targets of Immunotherapy in Breast Cancer and Pan-Cancer

**Figure 3:**
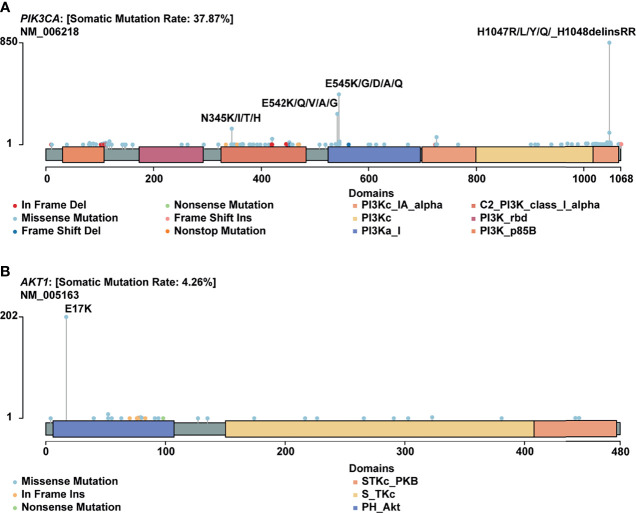
Mutational spectrum of specific genes. **(A)** Mutations across the *PIK3CA* gene, no corresponding neoantigen for E542K; **(B)** Mutations across the *AKT1* gene.

H1047R (*PIK3CA*), E545K (*PIK3CA*), E17K (*AKT1*), and N345K (*PIK3CA*) produced recurrent neoantigens and had a higher mutation frequency in the breast cancer cohort (**Figure 3**). Thus, we focused on these mutations and corresponding neoantigens.

**Figure 4:**
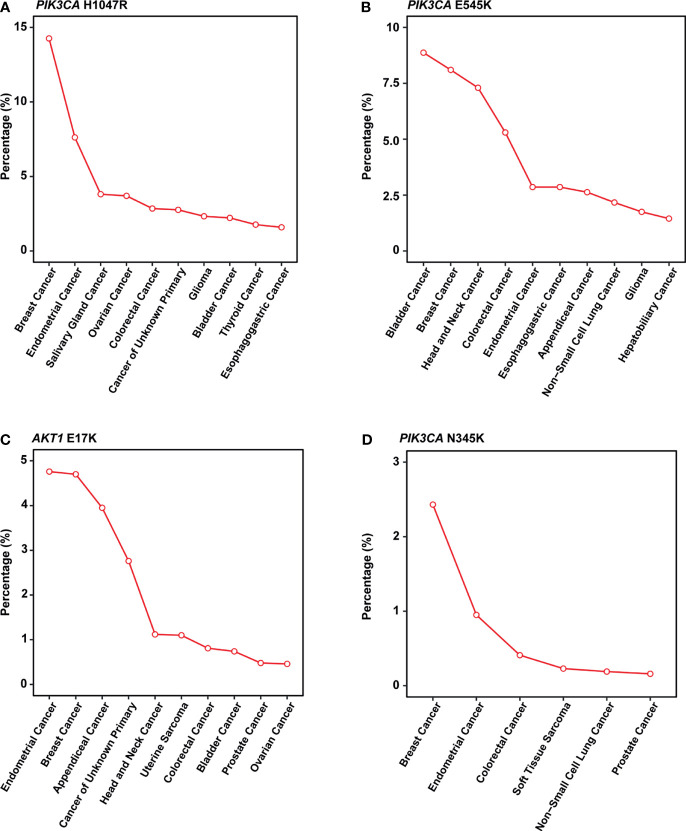
Mutation frequency across multiple cancer types in MSK-IMPACT cohorts. **(A-D)** Line graph showing the percentage of cancer. **(A)** *PIK3CA* H1047R; **(B)** *PIK3CA* E545K; **(C)** *AKT1* E17K; **(D)** *PIK3CA* N345K. All cancer types shown above should meet the following criteria: 1) with a total number of patients equals or exceed 50 in MSK-IMPACT cohort; 2) mutated frequency of the corresponding mutation should exceed 0; 3) if there are over 10 cancer types, show only the top 10 results with the highest frequency.

In this study, *PIK3CA* H1047R occurred in 14% of patients in our cohort, consistent with published research by Zehir *et al (* 22). Besides, Meyer and colleagues reported that this mutation in the luminal mammary epithelium could induce tumorigenesis (23). In many other cancer types, this mutation also showed a pretty high frequency (**Figure 4A**).

*PIK3CA* E545K is a hotspot mutation with first-line drugs (24). This mutation holds a frequency of about 8% in breast cancer, second to bladder cancer (**Figure 4B**). As for *PIK3CA* N345K, its mutation frequency is relatively low across all cancers as shown in **Figure 4D**. A case report suggested this mutation might be associated with the sensitivity of Everolimus (25).

*AKT1* E17K occurs in many solid tumors with a low frequency (**Figure 4C**). Compared to other *AKT1* mutations, E17K showed a higher occurrence (**Figure 3B**). In certain breast cancer patients, this mutation is most likely the driver mutation (26). Besides, a study has reported that *AKT1* E17K is a therapeutic target in many cancers (27).

## Discussion

In this study, we integrate 8 breast cancer research cohorts to depict the mutation panorama of breast cancer patients, which provide a reference for the genomics research of breast cancer and contributed to the in-depth study of clinical molecular typing of breast cancer patients. In addition, we predict a series of potential neoantigens based on the high-frequency mutation pairs after screening, which may serve as therapeutic targets for patients. *PIK3CA* and *TP53* are two highly mutated genes in breast cancer (28, 29). Our findings also showed this and further demonstrated they are mutually exclusive in mutations. Since TNBC is one of the most malignant breast cancers, we analyzed its mutation landscape and found *TP53* was a noteworthy gene with a very high frequency (79%). Besides, *PIK3CA* and *TP53* were differentially mutated in whatever subtypes of breast cancer, indicating their importance in the heterogeneity and development of breast cancer.

Common mutations present in at least 3 patients were used for neoantigen prediction. Since previous studies have proved that the common neoantigens may serve as immunotherapy targets (30, 31), we try to find out whether there are common neoantigens in breast cancer populations in this way. Indels were more capable to produce neoantigens than SNVs and can be recognized by more HLA subtypes. However, there are more SNVs than indels in patients, making SNVs the primary source of neoantigens in breast cancer. No statistical difference of indel-derived neoantigens was observed among all subgroups. In terms of SNV-derived neoantigens, age and HER2/ER/PR status are the vital influence factors.

As age increases, tumor mutational burden (TMB) increases accordantly (32). In our breast cancer cohort, the elder population (age>60) also held a higher non-synonym SNV background and a higher fraction of SNV-derived neoantigens. ER^-^, PR^-^ and HER^+^ patients held a higher SNV background but a lower fraction of patients with neoantigen. Thus, we infer that the SNV background in the age group might affect neoantigen production, but not in other subgroups. ER, PR, and HER2 status could be used as predictors of neoantigens for breast cancer patients.

H1047R (*PIK3CA*), E545K (*PIK3CA*), E17K (*AKT1*), and N345K (*PIK3CA*) were four hotspot mutations with derived neoantigens. Especially for *PIK3CA* H1047R, a driver mutation in breast cancer (33), was also reported as a neoantigen source in gastric cancer (30). *PIK3CA* E545K produced two different peptides and could be recognized by multiple HLA molecules, including HLA-A03:01, HLA-A11:01, and HLA-B57:01. We can infer that these mutations may serve as therapeutic targets for other cancers owing to their wide range in many cancers and recognition by multiple HLA molecules.

Here we focus on common neoantigens derived from high frequency mutations to benefit as many patients as possible. In spite of these important advantages, this study has several limitations. Due to the limitation of sample sources, the samples in the current data are mainly from European and American populations, which may make it difficult for the results to accurately describe the mutation characteristics of other populations such as Asia. In addition, although we have adopted a variety of stable and feasible bioinformatics methods, the currently predicted neoantigens still need further experimental validation.

## Conclusion

Based on the analysis of mutation data from eight breast cancer studies, we described the most complete mutation landscape of breast cancer so far. Forty-three HLA genotypes with high frequency in Chinese or TCGA cohort, and 738 non-silent somatic mutations were selected to predict the common neoantigens. The high-frequency mutations, including *PIK3CA* H1047R (14%), *PIK3CA* E545K (6.93%), *AKT1* E17K (3.27%) and *PIK3CA* N345K (2.20%), can be recognized by multiple HLA molecules, such as HLA-A11:01 and HLA-A03:01. These HLA genotypes are the dominant HLA subtypes in the Han Chinese and Americans, representing the commonality of neoantigens we identified among breast cancer patients. In conclusion, except for having constructed a comprehensive mutation landscape of breast cancer, we also have found a number of public neoantigens, which may contribute to the development of immunotherapy in breast cancer.

## Materials and Methods

### Genomic Data for Breast Cancer Patients

All somatic mutations, including single nucleotide variants (SNVs) and short insertion/deletion (indels), were obtained from the published datasets. The data comprise 5991 breast cancer patients from eight studies, covering several important studies, such as The Cancer Genome Atlas Program (TCGA). Clinical information is shown in **Table 3** and **Supplementary Table 4**. There is no need for additional informed consent because all data were from public databases with informed consent provided in the original studies.

**Table 3:** Summary of clinical information of 5991 patients from eight studies.

Characteristic	BCCRC (12)	BROAD (4)	IGR (16)	MBCproject	METABRIC (14)	MSK (15)	Sanger (13)	TCGA	Total
	(n=65)	(n=103)	(n=216)	(n=180)	(n=2509)	(n=1746)	(n=100)	(n=1072)	(n=5991)
**Age (years)**									
<=60	41 (63.1%)	86 (83.5%)	0 (0%)	107 (59.4%)	1163 (46.4%)	1333 (76.3%)	65 (65.0%)	593 (55.3%)	3388 (56.6%)
>60	22 (33.8%)	17 (16.5%)	0 (0%)	8 (4.4%)	1335 (53.2%)	413 (23.7%)	35 (35.0%)	479 (44.7%)	2309 (38.5%)
Unknown	2 (3.1%)	0 (0%)	216 (100%)	65 (36.1%)	11 (0.4%)	0 (0%)	0 (0%)	0 (0%)	294 (4.9%)
**Stage**									
0	0 (0%)	0 (0%)	0 (0%)	0 (0%)	24 (1.0%)	0 (0%)	0 (0%)	0 (0%)	24 (0.4%)
I	0 (0%)	11 (10.7%)	0 (0%)	11 (6.1%)	630 (25.1%)	469 (26.9%)	0 (0%)	181 (16.9%)	1302 (21.7%)
II	0 (0%)	73 (70.9%)	0 (0%)	27 (15.0%)	979 (39.0%)	512 (29.3%)	0 (0%)	608 (56.7%)	2199 (36.7%)
III	0 (0%)	19 (18.4%)	0 (0%)	21 (11.7%)	144 (5.7%)	370 (21.2%)	0 (0%)	246 (22.9%)	800 (13.4%)
IV	0 (0%)	0 (0%)	0 (0%)	51 (28.3%)	11 (0.4%)	381 (21.8%)	0 (0%)	18 (1.7%)	461 (7.7%)
Unknown	65 (100%)	0 (0%)	216 (100%)	70 (38.9%)	721 (28.7%)	14 (0.8%)	100 (100%)	19 (1.8%)	1205 (20.1%)
**ER Status**									
Positive	3 (4.6%)	44 (42.7%)	0 (0%)	94 (52.2%)	1825 (72.7%)	1372 (78.6%)	79 (79.0%)	0 (0%)	3417 (57.0%)
Negative	61 (93.8%)	28 (27.2%)	0 (0%)	19 (10.6%)	644 (25.7%)	329 (18.8%)	21 (21.0%)	0 (0%)	1102 (18.4%)
Unknown	1 (1.5%)	31 (30.1%)	216 (100%)	67 (37.2%)	40 (1.6%)	45 (2.6%)	0 (0%)	1072 (100%)	1472 (24.6%)
**PR Status**									
Positive	1 (1.5%)	40 (38.8%)	0 (0%)	83 (46.1%)	1040 (41.5%)	994 (56.9%)	60 (60.0%)	0 (0%)	2218 (37.0%)
Negative	63 (96.9%)	32 (31.1%)	0 (0%)	28 (15.6%)	940 (37.5%)	692 (39.6%)	40 (40.0%)	0 (0%)	1795 (30.0%)
Unknown	1 (1.5%)	31 (30.1%)	216 (100%)	69 (38.3%)	529 (21.1%)	60 (3.4%)	0 (0%)	1072 (100%)	1978 (33.0%)
**HER2 Status**									
Positive	0 (0%)	8 (7.8%)	14 (6.5%)	37 (20.6%)	247 (9.8%)	145 (8.3%)	30 (30.0%)	0 (0%)	481 (8.0%)
Negative	63 (96.9%)	47 (45.6%)	194 (89.8%)	71 (39.4%)	1733 (69.1%)	1248 (71.5%)	70 (70.0%)	0 (0%)	3426 (57.2%)
Unknown	2 (3.1%)	48 (46.6%)	8 (3.7%)	72 (40.0%)	529 (21.1%)	353 (20.2%)	0 (0%)	1072 (100%)	2084 (34.8%)

### Neoantigen Prediction

Mutations should be non-silent somatic mutations and identified in at least 3 breast cancer patients. Subsequently, These mutations, combined with 43 high-frequency HLA genotypes in Chinese and TCGA cohorts, were used to predict neoantigens through NetMHC (34), NetMHCpan (35), PSSMHCpan (36), PickPocket (37) and SMM (38). Criteria for neoantigen screening refers to our previous research (30).

### Statistical Analysis

We finished all statistical analyses in R-Studio (R version 3.6.0). The two R packages, maftools (39) and ggplot2 (40), were used for mutations analysis and visualization, respectively. Unless special instruction was given, *P* < 0.01 was considered significant.

## Data Availability Statement

The datasets presented in this study can be found in online repositories. The names of the repository/repositories and accession number(s) can be found in the article/**Supplementary Material**.

## Author Contributions

SZ, SL, LZ, and H-XS contributed to conception and design of the study. SZ and SL performed the statistical analysis. SZ, SL, LZ, and H-XS wrote sections of the manuscript. All authors contributed to the article and approved the submitted version.

## Conflict of Interest

The authors declare that the research was conducted in the absence of any commercial or financial relationships that could be construed as a potential conflict of interest.

## Publisher’s Note

All claims expressed in this article are solely those of the authors and do not necessarily represent those of their affiliated organizations, or those of the publisher, the editors and the reviewers. Any product that may be evaluated in this article, or claim that may be made by its manufacturer, is not guaranteed or endorsed by the publisher.

## Supplementary Material

Supplementary Figure 1 Landscape of the top 10 significantly mutated genes in all the breast cancer patients.

Supplementary Figure 2 The ratio of base conversion and transversion in mutations (including synonymous variants) and co-occurrence and exclusiveness of the top 20 mutated genes in breast cancer.

Supplementary Figure 3 The Mutation landscape of breast cancer patients with HER2^+^ and HER2^-^.

Supplementary Figure 4 Differentially mutated genes between HER2^+^ and HER2^-^ patients.

Supplementary Figure 5 The Mutation landscape and the differentially mutated genes between HR^+^ and HR^-^ patients.

Supplementary Figure 6 The mutation landscape of TNBC patients and the differentially mutated genes between TNBC and non-TNBC patients.

Supplementary Figure 7 Comparison of non-synonym SNV background within each subgroup, neoantigens among patients in different stages, and neoantigens derived from SNVs and indels.

Supplementary Table 1 The list of high-frequency HLA genotypes in Chinese and TCGA cohorts.

Supplementary Table 2 The list of SNV-derived neoantigens.

Supplementary Table 3 The list of indel-derived neoantigens.

Supplementary Table 4 The list of clinical information on samples in the previous eight studies of breast cancer.
