# Supplementary figures and images for: A Comprehensive Survey of Genomic Mutations in Breast Cancer Reveals Recurrent Neoantigens as Potential Therapeutic Targets
Source: Front Oncol. 2022 Mar 21;12:786438. doi: 10.3389/fonc.2022.786438 (PMC8978336; doi:10.3389/fonc.2022.786438)

Figure S1

A

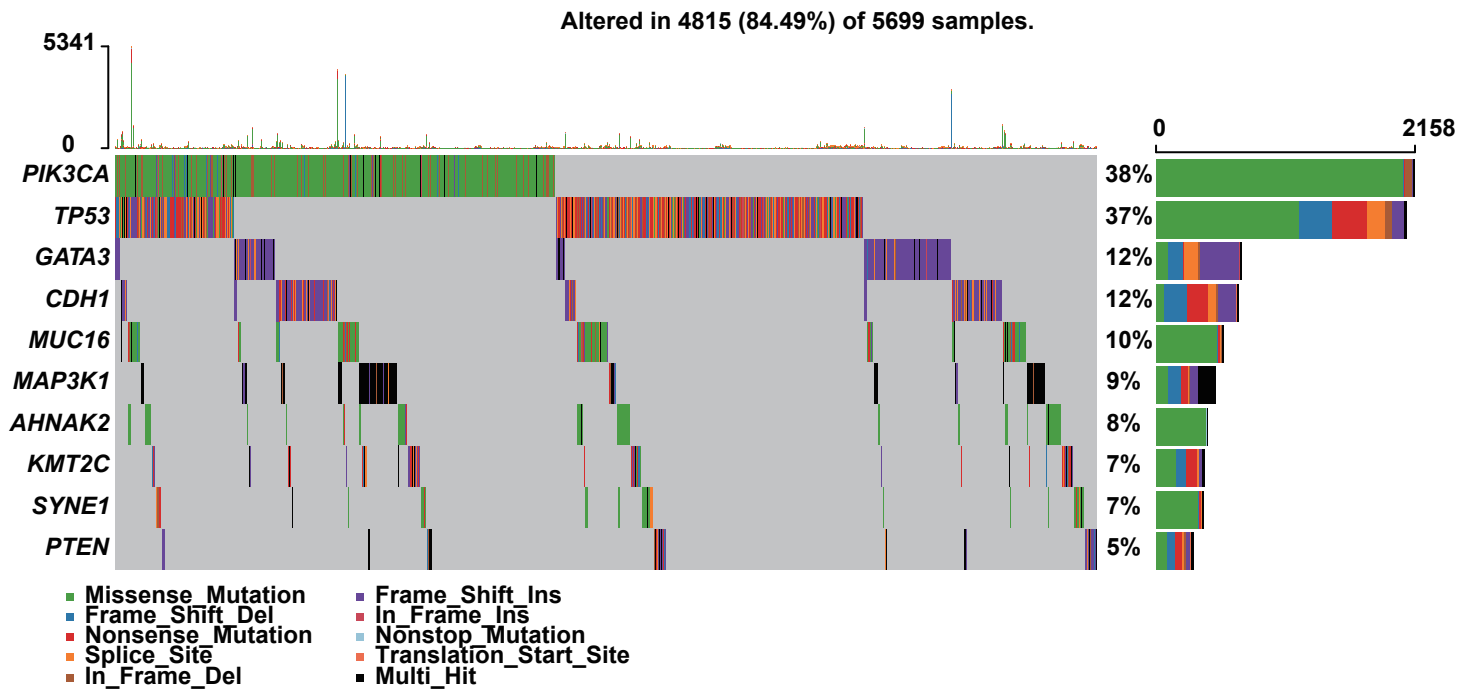

Supplement: Supplementary Figure 1 — Landscape of the top 10 significantly mutated genes in all the breast cancer patients. [file DataSheet_1.pdf]

Figure S2

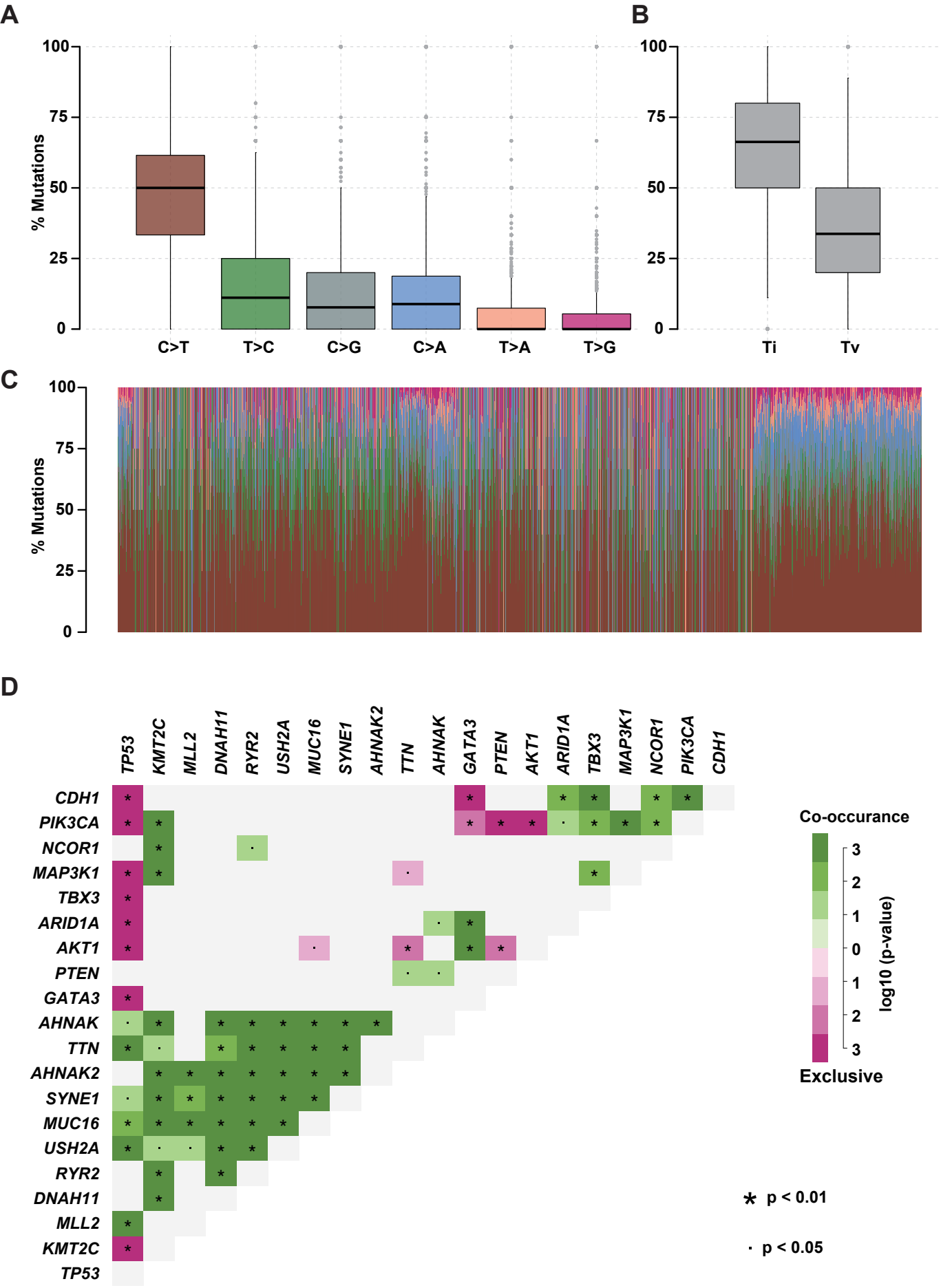

Supplement: Supplementary Figure 2 — The ratio of base conversion and transversion in mutations (including synonymous variants) and co-occurrence and exclusiveness of the top 20 mutated genes in breast cancer. [file DataSheet_2.pdf]

Figure S3

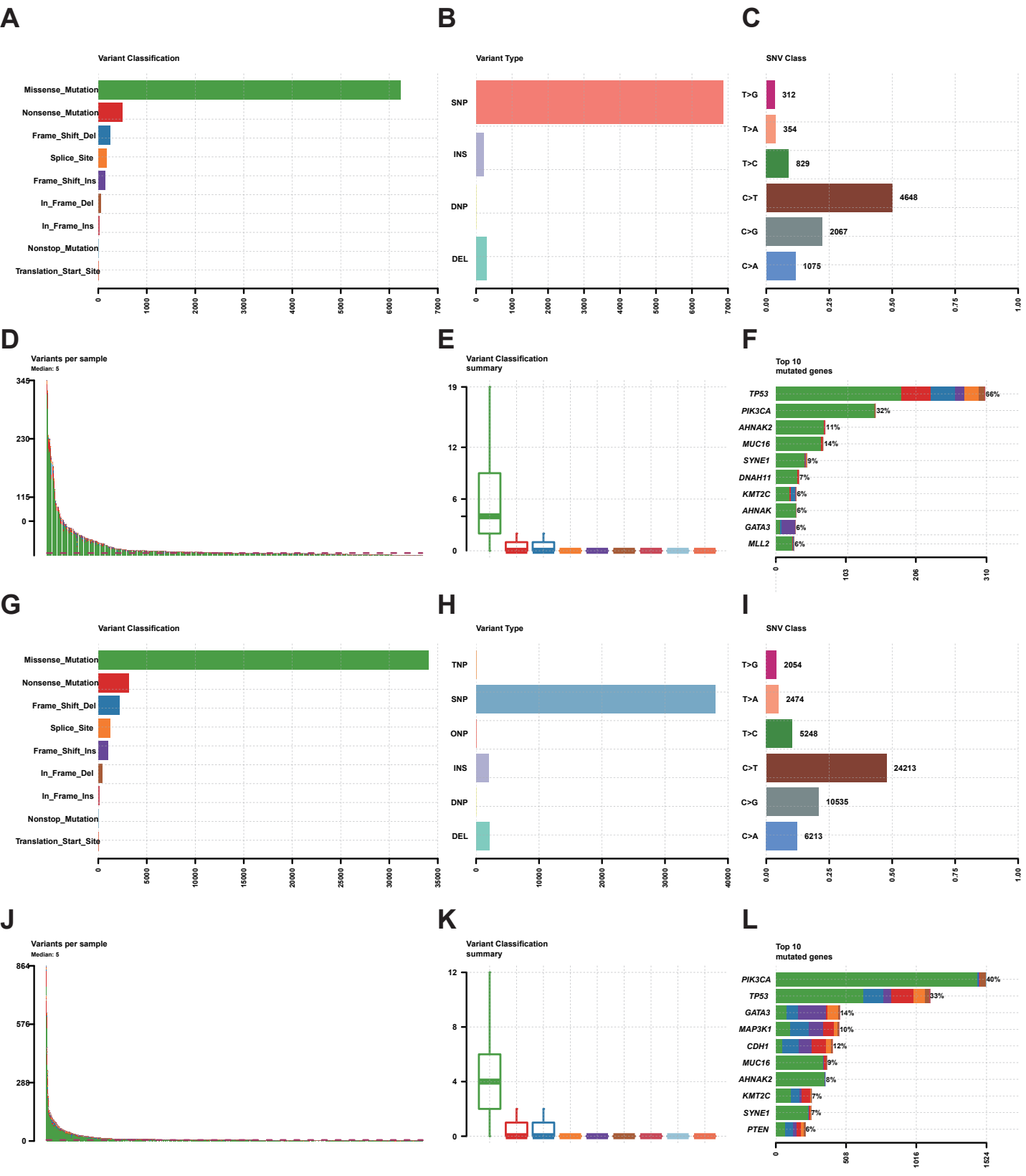

Supplement: Supplementary Figure 3 — The Mutation landscape of breast cancer patients with HER2+ and HER2-. [file DataSheet_3.pdf]

Figure S4

A

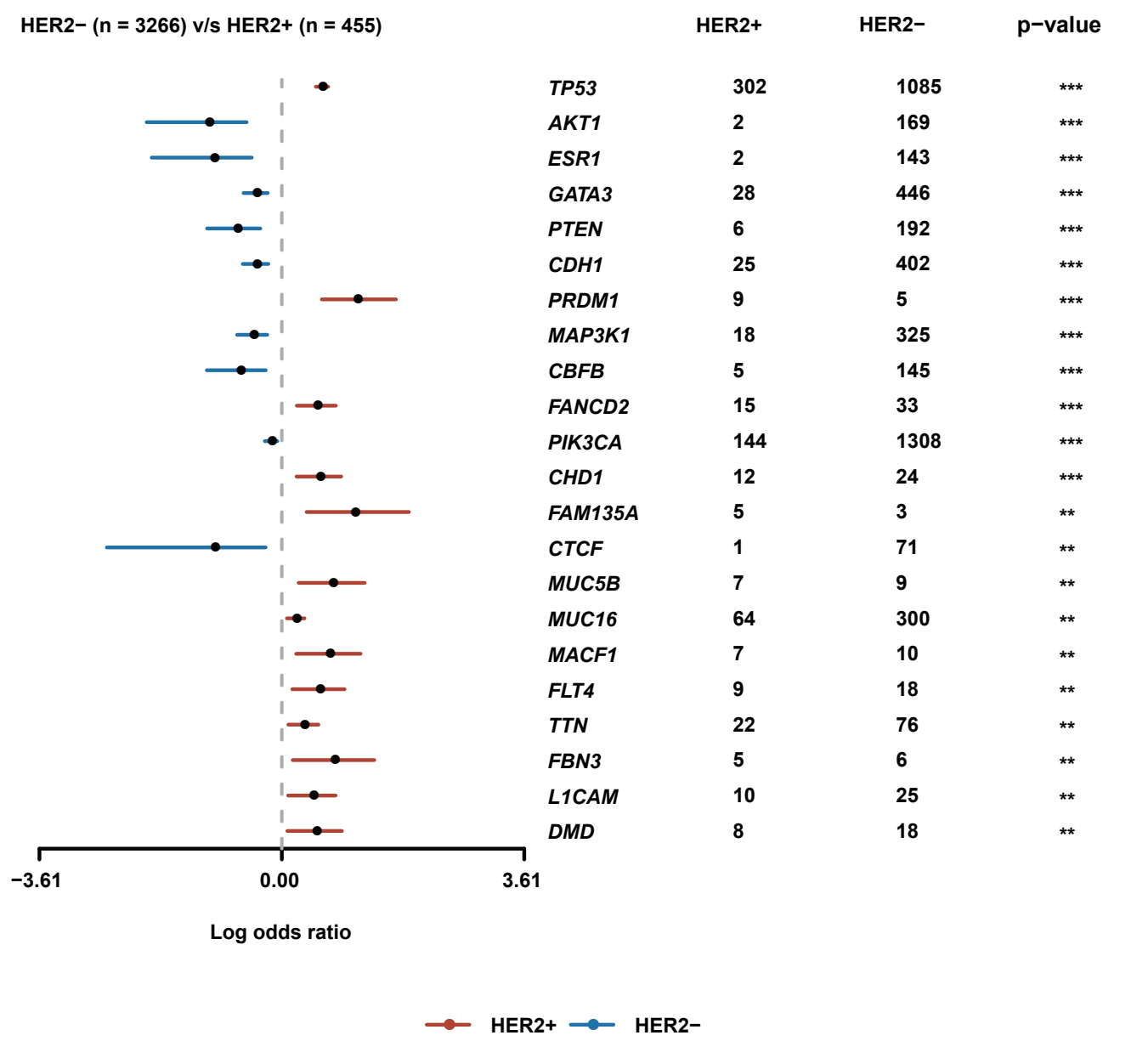

Supplement: Supplementary Figure 4 — Differentially mutated genes between HER2+ and HER2- patients. [file DataSheet_4.pdf]

Figure S5

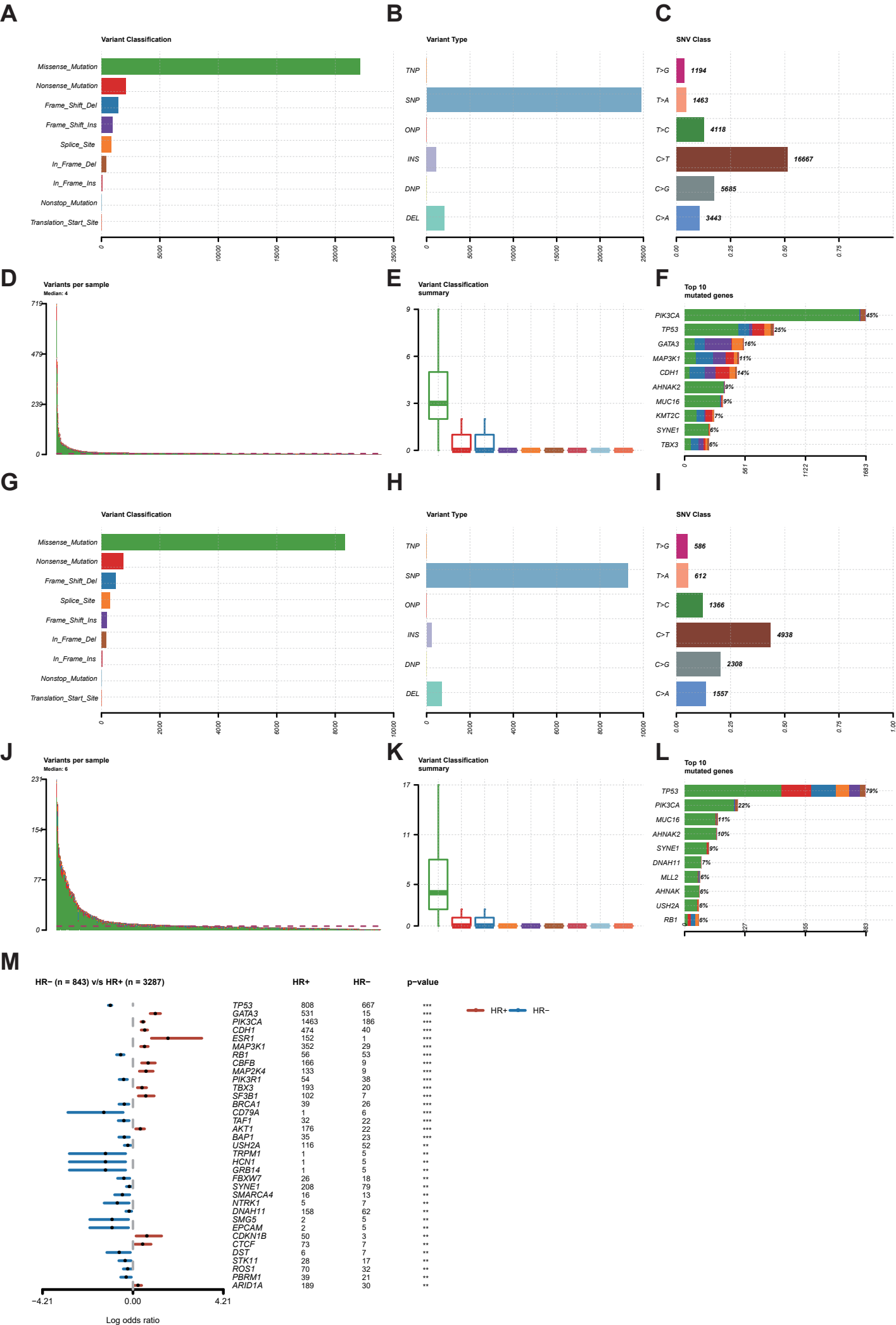

Supplement: Supplementary Figure 5 — The Mutation landscape and the differentially mutated genes between HR+ and HR- patients. [file DataSheet_5.pdf]

Figure S6

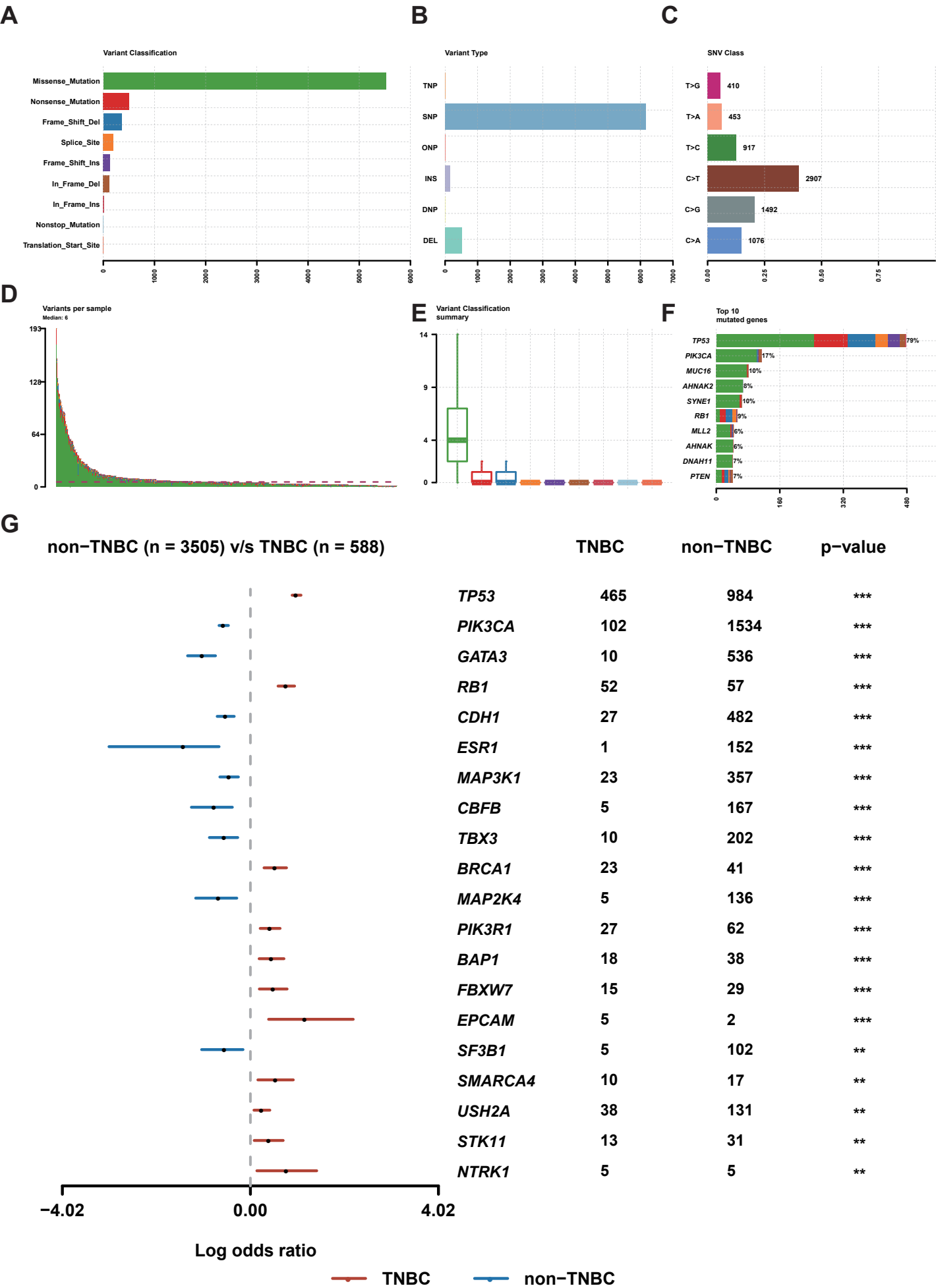

Supplement: Supplementary Figure 6 — The mutation landscape of TNBC patients and the differentially mutated genes between TNBC and non-TNBC patients. [file DataSheet_6.pdf]

Figure S7

A

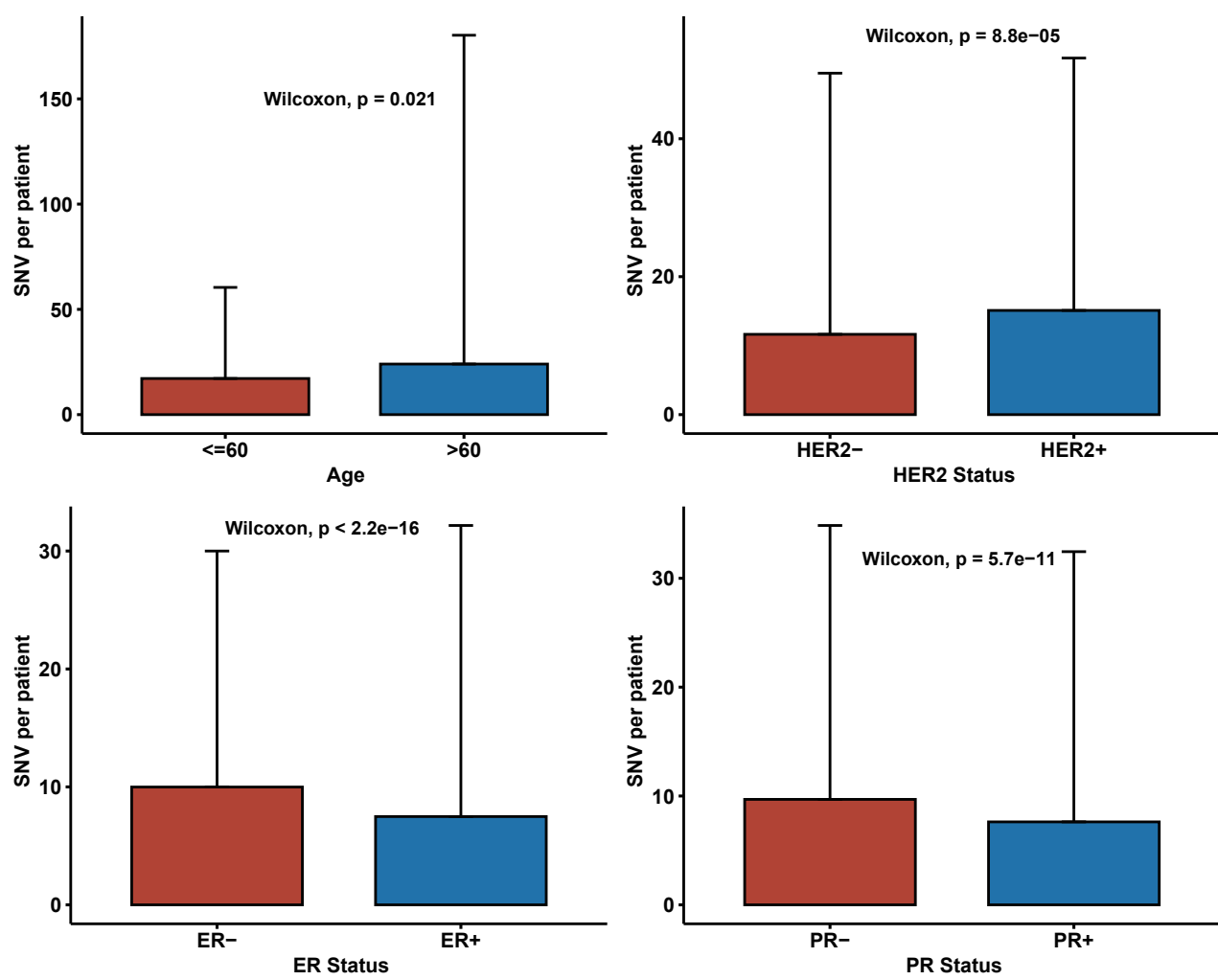

B

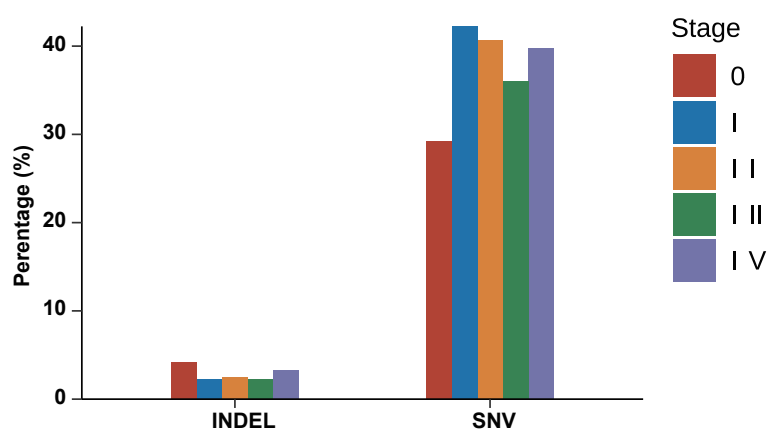

C

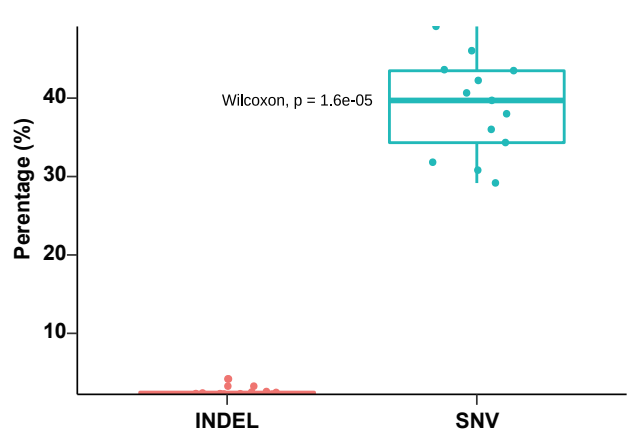

Supplement: Supplementary Figure 7 — Comparison of non-synonym SNV background within each subgroup, neoantigens among patients in different stages, and neoantigens derived from SNVs and indels. [file DataSheet_7.pdf]
